# Supplementary material for: TaNBR1, a Novel Wheat NBR1-like Domain Gene Negatively Regulates Drought Stress Tolerance in Transgenic Arabidopsis
Source: Int J Mol Sci. 2022 Apr 20;23(9):4519. doi: 10.3390/ijms23094519 (PMC9105663; doi:10.3390/ijms23094519)
Supplement: Supplementary file 1 [file ijms-23-04519-s001.zip › ijms-1668126-supplementary.pdf]

Article

TaNBR1, a novel wheat NBR1 like domains gene, negatively regulates drought stress tolerance in transgenic *Arabidopsis*

Liuping Chen[1], Qian Lv[1], Weibing Yang[1], Hui Yang[2], Qiaoyan Chen[3], Bingxin Wang[1], Yanhong Lei[1], and Yanzhou Xie[1*]

[1] State Key Laboratory of Crop Stress Biology in Arid Areas and College of Agronomy, Northwest A&F University, Xianyang 712100, China; chenliupinghist@163.com (L.C.); lvqianhist@163.com (Q.L.); jy_wheat@nwafu.edu.cn (W.Y.); wangbx@nwafu.edu.cn (B.W.); 2021060044@nwafu.edu.cn (Y.L.)

[2] Nanyang Academy of Agricultural Sciences, Nanyang, 473000, China；13721807607@163.com (H. Y)；

[3] College of Life and Science, Henan Institute of Science and Technology, Xinxiang, Henan 453000, China; CQY20100216@163.com (Q.C.);

* Correspondence: Yzxie@nwsuaf.edu.cn ; Tel: +86 29 8708 2022.

Table S1 Primer information used in these experiments

Primer name	primer sequence	Function or vector
TaNBR1 F1	GGACTCGCGCCATGAT	cDNA amplification
TaNBR1 R1	TTAATTAAGATATTCTCGCGCTACA	
gNBR1-1F	ATGATGCCTCAACGGGACA	gDNA amplification
gNBR1-1R	CCGCAGCCATCACATTCTAC	
gNBR1-2F	TTAATTAAGATATTCTCGCGCTACA	gDNA amplification
gNBR1-2R	ACCGTCGCCTCCAAAACC	
DNBR1F	CCAGGTCTAAGATGTCGG	qRT-PCR
DNBR1R	CACTGGTGGTGCCGAT	
YNBR1F	CGAGCTCAAGCTTCGAAATGATGCCTCAACGGGACAC	1302-EGFP
YNBR1R	CGTCGACTGCAGAATTCGAAATTAAGATATTCTCGCGCTACACAG	
AtActin F	TCGCTGACCGTATGAGCAAAG	qRT-PCR
AtActin R	TGTGAACGATTCCTGGACCTG	
P5CS1 F	GCGCATAGTTTCTGATGCAA	qRT-PCR

P5CS1 R	TGCAACTTCGTGATCCTCTG	
COR15AF	GGCCACAAAGAAAGCTTCAG	qRT-PCR
COR15AR	CTTGTTTGCGGCTTCTTTTC	
RD29A F	ATCACTTGGCTCCACTGTTGTTC	qRT-PCR
RD29A R	ACAAAACACACATAAACATCCAAAGT	
LEA14F	GTCATTCGATTCCGATCTGTGAGATC	qRT-PCR
LEA14R	CCCTACAACAGGAAGGTCGATGG	
RAB18F	TCGGTCGTTGTATTGTGCTT	qRT-PCR
RAB18R	CCAGATGCTCATTACACACTCATG	
NCED3F	ACCAACAAGAATGCCTTCCA	qRT-PCR
NCED3R	TAACAGAAACCAGCTGAGCTCGA	
TaACTIN F	CTCCCTCACAACAACCGC	qRT-PCR
TaACTIN R	TACCAGGAACTTCCATACCAAC	
